# Role of Myeloid Cells in Oncolytic Reovirus-Based Cancer Therapy

**DOI:** 10.3390/v13040654

**Published:** 2021-04-10

**Authors:** Vishnupriyan Kumar, Michael A. Giacomantonio, Shashi Gujar

**Affiliations:** 1 Department of Pathology, Dalhousie University, Halifax, NS B3H 4R2, Canada; vishnu.priyan@dal.ca (V.K.); M.Giacomantonio@dal.ca (M.A.G.); 2 Department of Microbiology and Immunology, Dalhousie University, Halifax, NS B3H 4R2, Canada; 3 Department of Biology, Dalhousie University, Halifax, NS B3H 4R2, Canada; 4 Beatrice Hunter Cancer Research Institute, Halifax, NS B3H 4R2, Canada

**Keywords:** reovirus, oncolytic virus, myeloid cell plasticity, tumor-associated macrophages, myeloid-derived suppressor cells, tumor microenvironment, immune checkpoint blockade, combination therapy

## Abstract

Oncolytic reovirus preferentially targets and kills cancer cells via the process of oncolysis, and additionally drives clinically favorable antitumor T cell responses that form protective immunological memory against cancer relapse. This two-prong attack by reovirus on cancers constitutes the foundation of its use as an anticancer oncolytic agent. Unfortunately, the efficacy of these reovirus-driven antitumor effects is influenced by the highly suppressive tumor microenvironment (TME). In particular, the myeloid cell populations (e.g., myeloid-derived suppressive cells and tumor-associated macrophages) of highly immunosuppressive capacities within the TME not only affect oncolysis but also actively impair the functioning of reovirus-driven antitumor T cell immunity. Thus, myeloid cells within the TME play a critical role during the virotherapy, which, if properly understood, can identify novel therapeutic combination strategies potentiating the therapeutic efficacy of reovirus-based cancer therapy.

## 1. Introduction

Cancers arise from genetically mutated cells that proliferate uncontrollably to form tumor masses, can infiltrate surrounding tissues, and also colonize distant niches via metastatic processes [1]. These oncogenic processes often facilitate the development of a highly suppressive tumor microenvironment (TME) that consists of resident and infiltrating cells, cytokines and chemokines, and an extracellular matrix (ECM) that surrounds the tumor [2]. The immune constituents of the TME contain a high degree of inter- and intra-tumoral heterogeneity and often support tumor growth, progression, and metastasis [2]. Indeed, a diverse array of immune evasion strategies aiding tumor survival are the hallmark of TME. 

**Figure 1:**
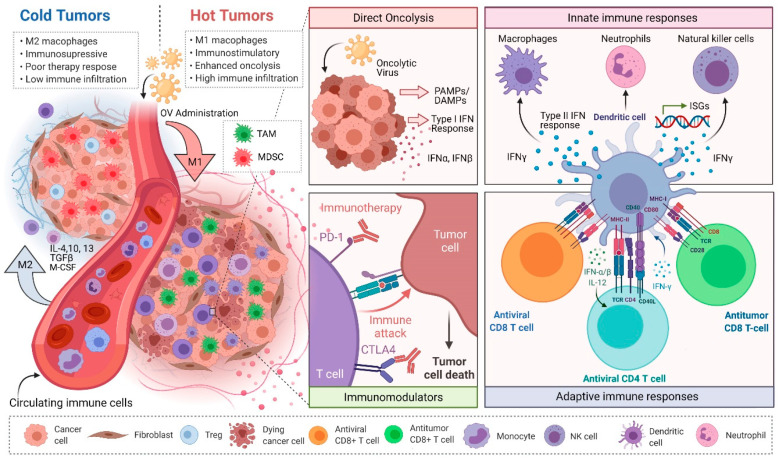
Overturning the tumor microenvironment (TME)-mediated immunosuppression using oncolytic virus (OV) immunotherapy. Administration of OVs can turn “cold” tumors “hot”, release otherwise inaccessible tumor antigens to be processed by antigen-presenting cells (APCs) via oncolysis and also drive the activation of innate immune cells. Further, OVs alone or in combination with ICB therapy can repolarize immunosuppressive immune cells, such as TAMs and MDSCs, to antitumor phenotype and support the development of antitumor immunity. Abbreviations: OV: Oncolytic virus; PD-1: Programmed cell death protein 1; CTLA-4: Cytotoxic T-lymphocyte-associated protein 4; IFN: Interferon; ISG: Interferon stimulating genes; IL: Interleukin; PAMPs: Pathogen-associated molecular patterns; DAMPs: Damage-associated molecular patterns; TGFβ: Transforming growth factor beta; CSF: Colony-stimulating factor.

The extensive heterogeneity of the TME and the resultant immunosuppression has been a major hurdle to the development of effective cancer treatments. Immune cells within the TME belong to both innate (e.g., myeloid cells, macrophages, dendritic cells, neutrophils, NK cells) and adaptive (e.g., T cells, B cells) arms of the immune system, and their infiltration into the tumor is highly dependent on the soluble factors present in the TME [2]. Based on the presence of these immune cells within the TME, tumors with little to no presence of immune cells are referred to as “cold” tumors, whereas “hot” tumors have an active presence of immune cells and are more likely to respond to immunotherapeutic approaches including adjuvant therapies and immune checkpoint blockade (ICB) [3] (Figure 1). Accordingly, therapeutic approaches that target both the cancer cells and the detrimental components of the TME have become expansive areas of research that have shown significant anticancer therapeutic potential. One such approach involves the use of oncolytic viruses (OVs) to simultaneously kill cancer cells and overturn tumor-associated immunosuppression [3,4,5].

OVs are derived from naturally occurring or genetically modified viruses, and preferentially infect and lyse cancer cells through a process of oncolysis [6]. It is now clear that most cancers harbor one or more intrinsic mechanisms (e.g., defective antiviral interferon response) through which the immune evasion is facilitated [7]. Interestingly, such immune defects within cancer cells also make them preferentially susceptible to infection by OVs. Additionally, multiple immunosuppressive strategies within the TME provide additional support towards the replication of OVs. Thus, as compared to non-transformed or “normal” cells, cancer cells and the TME represent a preferential niche enabling OV oncolysis [8]. 

Currently, many viruses are being investigated as OVs in preclinical and clinical testing, including reovirus [9]. While mammalian orthoreovirus (herein reovirus) is the most prevalent strain used in OV research, the oncolytic capacity of other zoonotic strains, such as avian orthoreovirus, are actively being explored for their potential use in patients with pre-existing immunity to mammalian orthoreoviruses [10,11]. Reovirus preferentially replicates in cancer or transformed cells and kills them via direct oncolysis [12]. Additionally, reovirus also overturns numerous immune evasion mechanisms present within the TME and facilitates the activation of antitumor CD8+ T cell responses [13,14,15,16]. Similar to other OVs, oncolytic reovirus-driven CD8+ antitumor T cell responses can target cancer cells—both at local and metastatic sites. Most importantly, reovirus-driven antitumor T cell immune response can resist a tumor challenge, suggesting the ability to protect against possible cancer relapse, and is clinically desired [17]. Unfortunately, while these reovirus-induced antitumor T cell responses are very promising, their efficacy is stunted by the TME. More specifically, the TME orchestrates the recruitment and differentiation of suppressive immune cells such as myeloid-derived suppressor cells (MDSCs) and tumor-associated macrophages (TAMs) that actively inhibit antitumor immune activities and enable cancer cells to escape immune-mediated elimination. Considering this significant impact, here we discuss the role of MDSCs and TAMs in the context of oncolytic reovirus-induced antitumor immunity, the detailed understanding of which will aid the optimum harnessing of reovirus-based cancer therapies. 

## 2. Myeloid Cell Plasticity and the TME 

**Figure 2:**
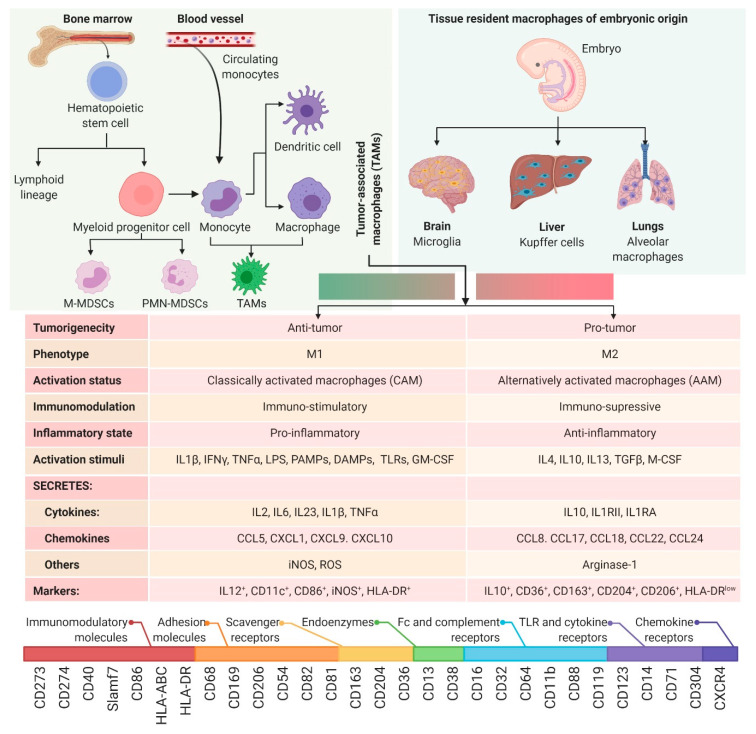
Ontogeny of macrophages. Top panel (left): Sources of macrophage recruitment, namely bone marrow and circulating monocytes; top panel (right): Various types of tissue resident macrophages; middle table: Characteristics of macrophage dichotomy; bottom: The spectrum of surface markers used to phenotype macrophages. Abbreviations: CAM: Classically activated macrophages; AAM: Alternatively activated macrophages; M-MDSC: Monocytic myeloid-derived suppressor cell; PMN-MDSC: Polymorphonuclear/granulocytic myeloid-derived suppressor cell; IL: Interleukin; IFN: Interferon; LPS: Lipopolysaccharide; PAMPs: Pathogen-activated molecular patterns; DAMPs: Damage-associated molecular patterns; TLR: Toll-like receptor; TNF: Tumor necrosis factor; CSF: Colony-stimulating factor; GM-CSF: Granulocyte-macrophage colony-stimulating factor; TGF: Transforming growth factor; iNOS: Nitric oxide synthase (inducible); ROS: Reactive oxygen species; CCL: Chemokine ligand; CXCL: C-X-C motif chemokine ligand; CD: Cluster of differentiation; HLA: Human leukocyte antigen; Slamf7: Signaling lymphocytic activation molecule F7.

The innate immune system represents an intricate network of highly diverse cells that collectively function to identify and eliminate pathogens from the body. Amongst these cells are myeloid cells, from which monocytes and macrophages originate (Figure 2). Monocytes and macrophages are found in the peripheral blood and are recruited to various tissues where they differentiate into their effector phenotype depending on the stimuli encountered in their microenvironment [18]. In response to a pathogen, macrophages are the first line of defense and can drive the initiation of an adaptive immune response through phagocytosis and antigen presentation [19]. Additionally, macrophages play a significant role in dictating the immune response either by maintaining homeostasis or through the secretion of immunogenic molecules including cytokines, chemokines, and complement factors [19]. However, the differentiation and physiological function of myeloid cells is context dependent and adaptable based on the stimuli encountered in their microenvironment [20]. 

### 2.1. M1 vs. M2 Macrophage Paradigm

Macrophage diversity is so extensive that it has been a significant cause of controversy within the field in regard to how different populations of macrophages should be classified [21,22,23,24]. While this debate still rages within the field of macrophage biology, the majority of researchers have adopted a model where macrophages are broadly classified into two types that represent opposing ends of a polarization spectrum: Classically activated (M1) and alternatively activated (M2) (Figure 2). M1 macrophages are polarized in response to pro-inflammatory stimuli such as interferon-gamma (IFNγ), lipopolysaccharide (LPS), granulocyte-macrophage colony-stimulating factor (GM-CSF), and toll-like receptor (TLR) stimulation. These cells develop a phenotype with functions that include pro-inflammatory cytokine production, endothelial cell activation, antiviral defense, and immune cell recruitment. Conversely, alternatively activated (M2) macrophages differentiate in response to anti-inflammatory stimuli such as interleukin-4 (IL-4) and macrophage colony-stimulating factor (M-CSF), generating a phenotype that specializes in tissue homeostasis, phagocytosis of apoptotic cells, and the production of anti-inflammatory cytokines (e.g., IL-4, -10, -13) [22,25,26]. In the context of cancer, M1 macrophages have antitumor properties, whereas M2 macrophages are immunosuppressive and are tumor-promoting [27]. Unfortunately, the current suboptimal levels of therapy responses to advanced treatments such as immune checkpoint blockade can be attributed to the complex heterogeneity within in the TME [28]. This complexity further makes it challenging to identify patients who will respond better to these drugs. A recent quest carried out in 98 pan-cancer patient data in the spirit of identifying biomarkers of value reiterated the prognostic significance of M1/M2 macrophage ratio [29,30]. It is also worth mentioning that the stimuli of polarization is a net result of the dynamic spectrum of various spatiotemporal signals present at the local environment [31]. While this nomenclature reflects a highly simplified model of myeloid cell plasticity, a comprehensive review of monocyte/macrophage dichotomy lies outside of the scope of this review and has been described elsewhere [32,33]. However, an understanding of how myeloid cell polarization is regulated in the context of the TME is critical in addressing the shortcomings of OV therapies.

### 2.2. Myeloid-Derived Suppressor Cells (MDSCs)

TME-associated growth factors, cytokines and chemokines mediate altered hematopoiesis that drives the generation of immunosuppressive MDSCs [34]. These chemotactic agents within TME also facilitate the migration of MDSCs. In animal models, the presence of C-C Motif Chemokine Ligand 2 (CCL2 or MCP-1), CCL3, CCL4, C-X-C ligand 12 (CXCL12), IL-6, IL-1β, M-CSF and vascular endothelial growth factor (VEGF) in the TME promoted MDSC infiltration [35,36]. MDSCs can be of polymorphonuclear/granulocytic phenotype (PMN-MDSC) or the monocytic phenotype (M-MDSC) [37]. Monocytes differentiate into macrophages and dendritic cells; whereas polymorphonuclear cells give rise to neutrophils, eosinophils, basophils, and mast cells [38]. The proliferation and differentiation of MDSCs are regulated by similar growth factors that regulate macrophage differentiation; namely M-CSF, G-CSF and GM-CSF [39]. Interestingly, however, MDSC recruitment and differentiation is believed to be much more complex than that of macrophages since signaling pathways are required to maintain their highly immature state [39]. Apart from the growth factors mentioned above, additional MDSC signaling pathways include a combination of TLR4 and IFNγ signaling, and the activation of the STAT3 pathway, amongst many more [40,41]. High frequencies of these cells clinically correlate with increased oncogenesis, metastasis and poor prognosis [37,42,43]. Moreover, the physical interaction between TAMs and MDSCs results in pro-tumorigenic Th2 immune response [44]. MDSCs also suppress antitumor T cell activities and promote angiogenesis [45]. Therefore, myeloid cell reprogramming strategies, alone or in combination with immunotherapies and anti-angiogenic strategies, will prove useful in combating these suppressive stimuli within the TME.

### 2.3. Tumor-Associated Macrophages (TAMs)

While often considered synonymous with M2 macrophages, TAMs can have characteristics of both M1 and M2 phenotypes depending on the type of cancer, stage of the tumor, and the TME [46]. Therefore, TAMs have a distinct phenotype from conventional macrophages, exhibiting significantly more immunosuppressive capacities than traditional M2 macrophages [46]. TAMs can originate from tissue resident macrophages or can develop from monocyte precursors in the blood [47]. The functional fate of TAMs is decided upon their recruitment into the TME in response to growth factors, cytokines and chemokines [27,48]. Signaling pathways known to drive TAM differentiation include VEGF, M-CSF, IL-4, CCL-2, -9, and -18 [48]. TAMs have been shown to make up to 50% of a tumors mass [49,50] and are therefore critical regulators of how a patient will respond to therapy [49]. In fact, TAM abundance in the TME has been shown to negatively correlate with survival in numerous cancers including breast cancer, renal cell carcinoma, glioblastoma, pancreatic cancer, head and neck cancer, lymphoma, and bladder cancer [22,31,32,51,52,53,54,55,56,57,58]. Moreover, TAMs have been shown to be instrumental for tumor relapse and metastasis in breast cancer [59,60,61]. Meta-analysis of clinical reports highlights that over 80% of the studies show a positive correlation between increased TAM density and poor patient prognosis [62]. CCL2 has also been reported to recruit TAMs which promotes metastasis in bone [63] and breast cancer [64] models. Interestingly, GM-CSF administration has been shown to counteract the immunosuppression and promote antitumor environment, likely by driving a more favorable pro-inflammatory TAM phenotype in addition to stimulating DC differentiation [65]. 

While TAMs and MDSCs are often similar in cellular functionality, they can be phenotypically distinguished from each other based on surface marker expression [48]. Additionally, while MDSCs are considered entirely tumor-promoting, TAMs have been shown to have both pro- and anti-tumor properties depending on the type of cancer, likely due to their previous roles as tissue resident macrophages prior to oncogenesis [48,66]. A better understanding of the intricacies involved in TME-TAM signaling will undoubtably provide more anticancer treatment options.

## 3. Reovirus-Based Dual-Prong Anticancer Actions and Myeloid Cells

Two main modes through which reovirus performs antitumor functions during virotherapy are: direct oncolysis and antitumor immunity. Following the therapeutic administration in cancer-bearing hosts, the efficacy of these reovirus-induced anticancer effects can be influenced by the myeloid cells within the TME.

### 3.1. Direct Oncolysis

While immunosuppression within the TME provides cancer cells with a competitive growth advantage, it also renders them conducive to reovirus infection and replication [67,68,69]. Here, the immunosuppressive components of the TME create an immune-privileged enclosure away from the reach of the properly functioning immune cells.

Like most viruses, productive reovirus infection is dependent on the host intracellular replication machinery [70]. The process of cellular transformation within cancer cells brings about dysregulation in metabolism and aberrant cell signaling pathways [1]. Reoviruses demonstrate an inherent propensity to infect transformed cells with such disrupted physiology [12]. The release of progeny viruses often leads to host cancer cell death, which makes it a lytic life cycle; the process is known as “oncolysis” [71]. Direct oncolysis by viral replication is facilitated using various signaling pathways involving TNFα, Fas ligand (FasL), TNF-related apoptosis-inducing ligand (TRAIL), ROS and others [72,73,74], and can lead to different types of cell death in different cancers [67,75]. Thus, reovirus-induced oncolysis of cancer cells involves multifaceted and complex interaction between cancer cells and surrounding tissues. In line with their immuno-modulatory effects, myeloid cells can influence reovirus-mediated oncolysis of cancer cells in a context-dependent manner. For example, M1 vs. M2 macrophages themselves bear different susceptibilities to virus infection and thus hold capacities to differentially regulate virus loads in the hosts [76]. Similarly, MDSCs themselves can carry replication-competent reovirus [77]. Nonetheless, the evidence on the role of myeloid cells on reovirus-mediated direct oncolysis, despite being important, is still in its infancy, and must be pursued further. These studies also should provide consideration for myeloid cell heterogeneity as the lack of information on the precise myeloid cell phenotypes has generated rather conflicting information for the OV field. For instance, while enhanced replication and oncolysis has been attributed to increased macrophage presence in oncolytic herpes simplex virus (HSV) infection of glioma, the depletion of macrophages in HSV-treated glioma was shown to result in higher titers of OV [78]. Similarly, in mesothelioma models, the presence of MDSCs hindered the ability of oncolytic modified vaccinia Tiantan (MVTT) to perform oncolysis and ablation of MDSCs restored the oncolytic efficacy of MVTT [79]. These findings are indicative that myeloid cells are central in orchestrating the OV-mediated oncolysis, and validate the notion that targeted reprogramming of MDSCs and TAMs will serve as a viable strategy to improve OV efficacy.

### 3.2. Reovirus-Induced Antitumor Immunity

Unfortunately, while the suppressive nature of the TME is actively involved in facilitating a productive reovirus infection [80], it also represents a daunting hindrance towards the initial activation and subsequent actions of antitumor CD8+ T cells [81]. Interestingly, reovirus has been shown to overturn many different immune evasion strategies present within the TME; examples include the promotion of antigen presentation and co-stimulation by antigen-presenting cells (including macrophages), recruitment of APCs and T cells within TME, subsequent trafficking to the lymph nodes, and production of pro-inflammatory cytokines. Cumulatively, these reovirus-driven immunological events drive the successful activation of protective antitumor CD8+ T cell responses. Unfortunately, the therapeutic injection of reovirus also promotes the recruitment of suppressive MDSCs which dampen the functions of antitumor CD8+ T cells [82]. Indeed, the inhibition of MDSC recruitment via C-C chemokine receptor type 2 (CCR2)-dependent mechanism or gemcitabine potentiates reovirus-induced antitumor effects [15,83]. Further, reovirus successfully inhibits the immunosuppressive activity of MDCSs in a TLR3-dependent manner and promotes antitumor CD8+ T cell responses [84]. Thus, the TME hosts a dynamic environment which although immunosuppressive can be harnessed to drive reovirus-induced antitumor benefits [6]. It should be noted that, similar to reovirus, the therapeutic implications for myeloid cells apply to other OVs as well. For example, oncolytic adenovirus and HSV infection led to increased infiltration of immunostimulatory macrophages in glioma [85,86], and exposure to oncolytic paramyxovirus resulted in skewing of macrophage phenotype towards antitumor capacity [87]. Further, oncolytic HSV in combination with gemcitabine has shown to effectively suppress MDSCs and result in tumor regression and enhanced oncolysis accompanied by antitumor CD4+ and CD8+ T cell responses [88]. Going forward, the context-dependent consideration for myeloid cell heterogeneity within OV-induced antitumor immune responses will be a key factor. Therefore, combination therapeutic strategies targeting the immunosuppressive aspects of the TME with specific regards to direct oncolysis and antitumor immunity will create optimal clinical outcomes from reovirus-based cancer therapy. 

## 4. Targeting Myeloid Cells to Improve Reovirus Therapy

Considering the immunosuppressive effects of the MDSCs and TAMs on antitumor immunity, currently many therapeutic strategies are being developed or tested to target these immunosuppressive myeloid cells, and thus are discussed below. It is worth mentioning that myeloid cells are merely one of many immunosuppressive cell types in the TME. For example, CD4^+^FoxP3^+^regulatory T cells (T_regs_) are highly immunosuppressive, and often play a central role in driving tumor immune evasion and suppression of antitumor immunity [89]. In fact, myeloid cells have been shown to drive the differentiation of T_regs_ in the TME through direct cell-cell interactions [90], further supporting the rational to therapeutically target MDSCs and TAMs during OV therapy. While a proper description of T_reg_ biology and function lies outside the scope of this review, this has been discussed extensively elsewhere [89,91,92]. Here, we discuss different combination therapy strategies aimed at targeting suppressive myeloid cells. Of note, many of the strategies are not yet used in conjunction with OVs; however, they are still discussed in anticipation of their future use in the context of OV-based cancer therapies such as reovirus. 

### 4.1. Blocking Recruitment

Blocking MDSCs and TAM recruitment can be an effective strategy to hinder tumorigenesis and alleviate immunosuppression. As CCL2 recruits TAMs and MDSCs that are positive for chemokine receptor CCR2 in TME [93,94], blockade of CCL2/CCR2 reverts the MDSC infiltration in in vivo models [95]. Accordingly, the ablation of CCR2 inhibition causes tumor relapse, favoring angiogenesis and metastasis in breast cancer models [96]. CCR2 inhibitors such as carlumab (CNTO 888), PF-04136309, MLN1202, BMS-813160, and CCX872-B are currently in clinical trials [97]. The CXCL-12-CXCR-4 axis also orchestrates the recruitment of TAMs via endothelial barrier into hypoxic tumor regions [98], and targeting CXCL-12-CXCR-4 axis alleviates tumor burden and metastatic susceptibility in breast, prostate, and ovarian cancer models by averting TAM infiltration [99,100]. Unfortunately, therapeutic administration of reovirus drives immediate recruitment of MDSCs in TME, which, if inhibited, potentiates antitumor CD8+ T cell responses [15]. 

### 4.2. Depleting Macrophage Populations in the TME 

TAMs can be directly depleted by triggering apoptosis [101]. Bisphosphonates preferentially target phagocytic cells such as TAMs and elicit myeloid cell cytotoxicity [102]. Zoledronate, a third-generation bisphosphonate displays cytotoxicity towards matrix metalloproteinase-9 (MMP9)-expressing TAMs and enhances antitumor activity of macrophages by reprogramming monocyte differentiation towards a pro-inflammatory phenotype [103]. Trabectedin, a chemotherapeutic agent, selectively elicits cell death in monocytes including TAMs through the activation of the caspase-8 cascade via TRAIL receptors [104]. Retrospective analysis of data from 34 patients who received trabectedin-based chemotherapy revealed that 56% of patients were found to have a reduction in monocytes in the tumor [104]. Trabectedin administration prior to oncolytic HSV injection has been shown to deplete intratumoral myeloid cells including macrophages [105]. Importantly, Trabectedin prevented the increase in intratumoral infiltration of these cells upon oncolytic HSV injection [105]. The depletion of TAMs also markedly increased the antitumor efficacy of oncolytic HSV by significantly altering the TME [105] in many models including Ewing Sarcoma and glioblastoma [106]. These and similar other myelolytic treatments are promising strategies to synergistically increase OV therapy efficacy.

### 4.3. Reprogramming Metabolism 

In macrophages, growth-factor-driven metabolic rewiring results in phenotypic alterations that enable them to adapt to their environment and perform their immune effector functions. For example, in response to pro-inflammatory stimuli, M1 macrophages disrupt the tricarboxylic acid (TCA) cycle at two points—after citrate and after succinate—driving fatty acid synthesis (FAS) and IL-1β production which are central to their pro-inflammatory phenotype [107,108,109,110]. Conversely, in the context of anti-inflammatory stimuli, M2 macrophages have an intact TCA cycle that favors mitochondrial oxidative phosphorylation (OXPHOS), resulting in a greater yield of ATP [107,108,109,110]. Accordingly, inhibition of ATP synthesis in these macrophages using ATP synthase inhibitor, oligomycin, or hexokinase inhibitor, 2-deoxyglucose suppresses anti-inflammatory gene and marker expression, and overall function [111,112]. Therefore, it is not surprising that TME-associated metabolic aberrations affect the functional attributes of TAMs. Thus, targeted metabolic reprogramming of macrophages in the context of metabolic perturbations within the TME represent next frontiers in harnessing OV cancer therapy efficacies.

### 4.4. Reprogramming Cellular Signaling

OVs in combination with strategies aimed to overturn the immunosuppressive cues in the TME are effective tools to reprogram myeloid cells towards an antitumor phenotype. TAMs and MDSCs can be re-educated to be tumoricidal using strategies such as, but not limited to CSF1/CSF1R blockades, TLR agonists, PI3Kγ inhibitors, CD40 agonists, and Class IIa histone deacetylase (HDAC) inhibitors [113]. The surface receptors of macrophages that facilitate antibody-dependent cellular cytotoxicity/phagocytosis (ADCC/ADCP) are attractive targets. Tumor cells express CD47 which recognize signal regulatory protein alpha (SIRPα) receptor on macrophages to help them escape immune surveillance [114]. However, this can be overcome by employing anti-SIRPα antibody to elicit macrophage-dependent cellular phagocytosis [115]. TLRs are also capable of directing TAMs towards a pro-inflammatory antitumor phenotype, when subjected to TLR agonists. TLR7/TLR8 agonists have been shown to counteract subcutaneous melanoma in vivo, in concert with ICB therapy. CSF1R inhibition when combined with oncolytic adenovirus and anti-PD-1 antibody enhances tumor regression and confers survival advantage to mouse models of colon cancer [116]. An oncolytic adenovirus engineered with TLR agonistic immunostimulatory-islands can overcome myeloid cell-mediated immunosuppression and elicit effective antitumor T cell responses [117]. Arming an oncolytic adenovirus with CD40L successfully repolarizes M2 macrophages, induces oncolysis and promotes intratumoral T cell infiltration and expansion [118]. These arguments underline the potential of reprogramming TAMs and MDSCs for enhancing OV efficacy.

Considering the central role of GM-CSF in myeloid cell biology, OVs have been engineered to express this cytokine. Recently, Kemp et al., successfully generated reoviruses that express murine and human GM-CSF (rS1-mmGMCSF and rS1-hSGMCSF, respectively). In a murine model of pancreatic cancer, intratumoral treatment with rS1-mmGMCSF resulted in an increase in T cell activation at distant metastatic tumor sites [119]. This supports the notion that reprogramming the TME by targeting myeloid populations improves the ability of reovirus to drive an antitumor T cell response. With respect to other OVs, Pexa-Vec (also known as JX-594), a vaccinia virus, has been engineered to express GM-CSF in order to repolarize infiltrating MDSCs and TAMs [120]. Trials using Pexa-Vec have demonstrated safety and showed antitumor activities in colorectal, hepatocellular and pediatric cancer [121,122]. Similarly, RP1, an oncolytic herpes simplex virus (HSV) armed with GM-CSF encoding region [123], has reported increased immune infiltration and robust CD8+ T cell responses [124]. The first approved [125] OV for clinical use namely T-VEC (Talimogene laherparepvec) [126], is armed with one cassette encoding human GM-CSF [127]. A list of clinical trials which use OVs in combination with immunomodulating therapies aimed to reprogram MDSCs and TAMs in various cancers can be found in Table 1.

### 4.5. Immune Checkpoint Blockade (ICB)

Blockade of immune checkpoint proteins namely, programmed cell death protein 1 (PD-1), its ligand PD-L1, and cytotoxic T-lymphocyte-associated protein 4 (CTLA-4) in combination with reovirus have shown potential in improving efficacy of reovirus therapy (Table 2). This therapeutic approach is not restricted to reovirus, as other OVs are also increasingly explored to function in synergy with ICB therapies. Table 3 lists the clinical trials currently underway which employ OVs in combination with ICB therapies for various cancers. Within these mainstream immune checkpoints, PD-L1 is known to be expressed on MDSCs and TAMs, and thus ICB with anti-PD-L1 strategies can directly affect myeloid cell functions [128]. While PD-1 and CTLA-4 are known to be expressed mainly on effector immune cells (e.g., T and NK cells), the blockade of both these molecules have shown to reprogram myeloid biology and to bear therapeutic implications [129,130]. Interestingly, MDSCs have also been identified to be a key mediator in conferring resistance to cancer immunotherapy [131]. While anti-PD-L1 and anti-PD-1 therapy are often discussed interchangeably, literature is beginning to show differences in patient responses. Accordingly, research has shown a differential response to PD-1 blockade and PD-L1 blockade in myeloid cells, where the latter leads to more robust inflammatory responses including IL-18 production and inflammasome activation [132]. An understanding of how these responses differ in the context of reovirus therapy is required and will lead to a better approach to combination therapy. 

**Figure 3:**
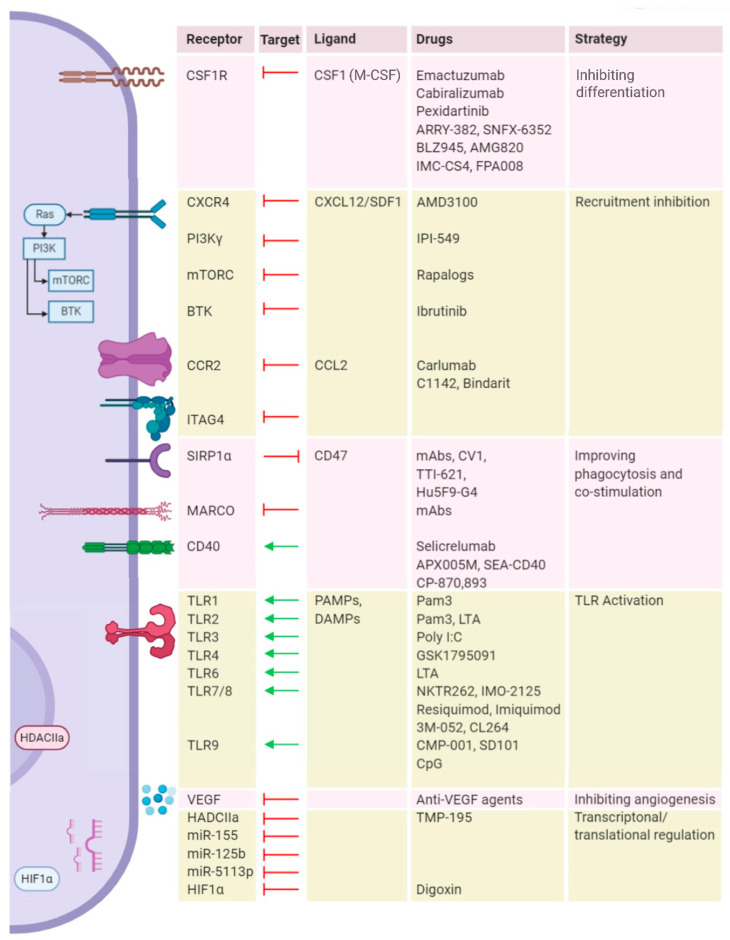
Current landscape of macrophage repolarization strategies. Various receptors, secretory molecules and regulatory pathways that skew macrophages to M2 state are lined on the left, and the respective therapeutic targets, their ligands, targeting strategies, and therapeutic agents are listed in the corresponding table on the right. Abbreviations: CSF1R: Colony-stimulating factor 1 receptor; CSF1: Colony-stimulating factor 1; M-CSF: Macrophage colony-stimulating factor; CXCR4: C-X-C chemokine receptor type 4; CXCL12: C-X-C motif chemokine ligand 12; SDF1: stromal cell-derived factor 1 (SDF1); PI3K: Phosphoinositide 3-kinase; mTORC: Mammalian target of rapamycin complex; BTK: Bruton’s tyrosine kinase; CCR2: C-C chemokine receptor type 2; CCL2: C-C Motif chemokine ligand 2; ITAG: Integrin alpha gamma; SIRP1α: signal regulatory protein alpha; MARCO: Macrophage receptor with collagenous structure; TLR: Toll-like receptor; CD: Cluster of differentiation; PAMPs: Pathogen-activated molecular patterns; DAMPs: Damage-associated molecular patterns; VEGF: Vascular endothelial growth factor; HDACIIa: Histone deacetylase class IIa; HIF1α: Hypoxia-inducible factor 1-alpha.

In addition to these mainstream ICBs, a range of other immune targets have been identified for reprogramming these suppressive myeloid cell types towards an antitumor phenotype. Here, CSF1R is the most widely explored target followed by TLR-4, TLR-7, TLR-8, TLR-9, CD40, CD47, and SIRPα [133,134,135]. Other targets such as BTK, CCR2, and RIP are also getting appreciated [99,136,137,138]. A list of clinical trials that exploit the targets for macrophage repolarization in synergy with ICB are listed in Table 4 and a list of immunological targets on macrophages are represented in Figure 3.

## 5. Conclusions

Reovirus represents a promising oncolytic agent, and its ability to drive an adaptive antitumor T cell response makes it an increasingly attractive immunotherapeutic. However, the efficacy of reovirus-based therapies remains suboptimal due to the highly suppressive effects of the TME. In particular, tumor-infiltrating myeloid cell populations such TAMs and MDSCs significantly contribute to these immunosuppressive effects using a variety of processes, including the production of anti-inflammatory cytokines, metabolic dysregulation, and T cell suppression. These TAM- and MDSC-based mechanisms hinder the efficacy of reovirus therapy but can be therapeutically targeted via blocking myeloid cell recruitment, depleting myeloid cell populations in the TME, reprogramming tumor or myeloid cell metabolism, and correcting aberrant cellular signaling pathways. The efficacy of many of these approaches is yet to be described in a reovirus-specific manner; therefore, further investigations testing the combination of reovirus and the TAM-/MDSC-targeting strategies promise to develop novel anticancer options.

## Figures and Tables

**Table 1:** List of clinical trials using OVs in combination with immunomodulatory strategies for various cancers.

Trial	Virus Type	OV Agent	Immunomodulatory Therapy		Phase (s)	Cancer (s)
			Agent	Description		
NCT03747744	HSV	T-Vec	CD1c (BDCA-1)+ myDC	CD1c+ myeloid dendritic cells	I	Melanoma
NCT02197169	Adenovirus	DNX-2401	IFNγ	Pro-inflammatory	I	Glioblastoma or Gliosarcoma
NCT02143804	Adenovirus	CG0070	GM-CSF (encoded)	M1 polarizer	II	Bladder Cancer, High Grade, Non-Muscle Invasive
NCT00625456	Vaccinia	RACVAC (JX-594)	GM-CSF (encoded)	M1 polarizer	I	Melanoma, Lung Cancer Renal Cell Carcinoma, Squamous Cell Carcinoma of the Head and Neck, Neuroblastoma
NCT01169584	Vaccinia	RACVAC (JX-594)	GM-CSF (encoded)	M1 polarizer	I	Rhabdomyosarcoma, Lymphoma Wilm’s Tumor, Ewing’s Sarcoma
NCT04725331	Vaccinia	BT-001	Anti CTLA-4 mAb (encoded)	ICB	I, II	Solid Tumor, Adult Metastatic Cancer Soft Tissue Sarcoma, Merkel Cell Carcinoma, Melanoma, Triple Negative Breast Cancer Non-Small Cell Lung Cancer
NCT04050436	HSV	RP1	GM-CSF (encoded)	M1 polarizer	II	Squamous Cell Carcinoma
			Cemiplimab (combination)	PD-1		
NCT03767348	HSV	RP1	GM-CSF (encoded)	M1 polarizer	II	Melanoma, NSCLC
			Nivolumab (combination)	PD-1	II	
NCT00554372	Vaccinia	JX-594	GM-CSF (encoded)	M1 polarizer	II	Hepatocellular Carcinoma
NCT00629759	Vaccinia	JX-594	GM-CSF (encoded)	M1 polarizer	I	Liver neoplasms
NCT04521764	Measles	MV	NAP (encoded)	Secretes neutrophil activating protein	I	Breast cancer

**Table 2:** List of clinical trials using reovirus in combination with immune checkpoint blockade therapies for various cancers.

Trial	Reoviral Agent	ICB therapy		Phase (s)	Cancer (s)
		Agent	Target		
NCT03723915	Pelareorep	Pembrolizumab	PD-1	II	Pancreatic adenocarcinoma, Pancreatic cancer
NCT03605719	Pelareorep	Nivolumab	PD-1	I	Recurrent Plasma Cell Myeloma
NCT04102618	Pelareorep	Atezolizumab	PD-L1	I	Breast cancer
NCT02620423	Reolysin	Pembrolizumab	PD-1	I	Pancreatic Adenocarcinoma
NCT04445844	Pelareorep	Retifanlimab	PD-1	II	Breast cancers
NCT04215146	Pelareorep	Avelumab	PD-L1	II	Metastatic breast cancer

**Table 3:** List of clinical trials using OVs in combination with immune checkpoint blockade therapies for various cancers.

Trial	Virus Type	OV Agent	ICB Therapy		Phase (s)	Cancer (s)
			Agent	Target		
NCT03004183	Adenovirus	ADV/HSVtk	Pembrolizumab	PD-1	II	Metastatic NSCLC, Metastatic TNBC
NCT02798406	Adenovirus	DNX-2401	Pembrolizumab	PD-1	II	Glioblastoma, Gliosarcoma
NCT03003676	Adenovirus	ONCOS-102	Pembrolizumab	PD-1	I	Advanced/unresectable melanoma progressing after PD-1 blockade
NCT03408587	Coxsackie	CAVATAK	Ipilimumab	CTLA-4	Ib	Uveal Melanoma with Liver Metastases
NCT02565992	Coxsackie	CAVATAK	Pembrolizumab	PD-1	I	Advanced Melanoma
NCT02824965	Coxsackie	CAVATAK	Pembrolizumab	PD-1	I, II	Advanced NSCLC
NCT03153085	HSV	HF10 (TBI-1401)	Ipilimumab	CTLA-4	II	Unresectable/Metastatic Melanoma
NCT02272855	HSV	HF10 (TBI-1401)	Ipilimumab	CTLA-4	II	Unresectable/Metastatic Melanoma
NCT03259425	HSV	HF10 (TBI-1401)	Nivolumab	PD-1	II	Resectable Stage IIIB/C, IV Melanoma
NCT01740297	HSV	T-Vec	Ipilimumab	CTLA-4	Ib, II	Unresected Stage IIIb/IV melanoma
NCT02263508	HSV	T-Vec	Pembrolizumab	PD-1	Ib, III	Unresectable Stage IIIb/IV Melanoma
NCT02626000	HSV	T-Vec	Pembrolizumab	PD-1	Ib, III	Recurrent/Metastatic HNSCC
NCT02879760	Maraba Virus	MG1-MAGEA3	Pembrolizumab	PD-1	I, II	Previously treated NSCLC
NCT03206073	Vaccinia	Pexa Vec	Durvalumab	PD-L1	I, II	Refractory Colorectal Cancer
			Tremelimumab	CTLA-4		
NCT02977156	Vaccinia	Pexa Vec	Ipilimumab	CTLA-4	I	Metastatic/Advanced Solid Tumors
NCT03071094	Vaccinia	Pexa Vec	Nivolumab	PD-1	I, IIa	Advanced HCC
NCT04185311	HSV	T-Vec	Ipilimumab	CTLA-4	I	Localized Breast Cancer
			Nivolumab	PD-1		
NCT03889275	Newcastle disease virus	MEDI5395	Durvalumab	PD-L1	I	Advanced Solid Tumors
NCT04301011	Vaccinia	TBio-6517	Pembrolizumab	PD-1	I, II	Triple Negative Breast Cancer Microsatellite Stable Colorectal Cancer
NCT04735978	HSV	RP3	Anti-PD-1 mAb	PD-1	I	Advanced Solid Tumor
NCT04348916	HSV	ONCR-177	Pembrolizumab	PD-1	I	Advanced Solid Tumors
NCT03294083	Vaccinia	Pexa Vec	Cemiplimab	PD-1	I, II	Renal Cell Carcinoma
NCT04755543	HSV	OH2	LP002	PD-1	I	Digestive System Neoplasms
NCT04386967	HSV	OH2	Keytruda	PD-1	I, II	Solid Tumors, Melanoma
NCT04616443	HSV	OH2	HX008	PD-1	I, II	Melanoma
NCT03866525	HSV	OH2	HX008	PD-1	I, II	Solid Tumors, Gastrointestinal Cancer
NCT03206073	Vaccinia	Pexa-Vec	Durvalumab	PD-L1	I, II	Colorectal Neoplasms
			Tremelimumab	CTLA-4		
NCT04665362	Alphavirus	M1	SHR-1210	PD-1	I	Advanced/Metastatic Hepatocellular Carcinoma
NCT04685499	Adenovirus	OBP-301	Pembrolizumab	PD-1	II	HNSCC

**Table 4:** List of clinical trials targeting TAMs in combination with immune checkpoint blockade therapies for various cancers.

Trial	TAM-Directed Agent		ICB Therapy		Phase (S)	Cancer (S)
	Agent	Target	Agent	Target		
NCT02323191	Emactuzumab	CSF1R	Atezolizumab	PD-L1	I	Locally advanced or metastatic solid tumors
NCT02880371	ARRY-382	CSF1R	Pembrolizumab	PD-1	I/II	Advanced solid tumors
NCT02777710	Pexidartinib	CSF1R	Durvalumab	PD-L1	I	Colorectal cancer; Pancreatic cancer; Metastatic cancer; Advanced cancer
NCT03238027	SNFX-6352	CSF1R	Durvalumab	PD-L1	I	Solid tumor; Metastatic tumor; Locally advanced malignant neoplasm; Unresectable malignant neoplasm
NCT02829723	BLZ945	CSF1R	PDR001	PD-1	I/II	Advanced solid tumors
NCT03158272	Cabiralizumab	CSF1R	Nivolumab	PD-1	I	Advanced malignancies
NCT02713529	AMG820	CSF1R	Pembrolizumab	PD-1	I/II	Pancreatic cancer; Colorectal cancer; Non-small cell lung cancer
NCT03123783	APX005M	CD40	Nivolumab	PD-1	I/II	Non-small cell lung cancer; Metastatic melanoma
NCT02304393	Selicrelumab	CD40	Atezolizumab	PD-L1	I	Solid tumors
NCT02637531	IPI-549	PI3Kγ	Nivolumab	PD-1	I	Advanced solid tumor; non-small cell lung cancer; melanoma; breast cancer
NCT02890368	TTI-621	SIRPα	Nivolumab	PD-1	I	Solid tumors; melanoma; merkel-cell carcinoma; squamous cell carcinoma; breast carcinoma
			Pembrolizumab	PD-1		
			Atezolizumab	PD-L1		
			Durvalumab	PD-L1		
NCT03530683	TTI-621	SIRPα	Nivolumab	PD-1	I	Lymphoma; myeloma
			Pembrolizumab	PD-1		
NCT03681951	GSK3145095	RIP	Pembrolizumab	PD-1	I/II	Neoplasms; pancreatic
NCT03435640	NKTR262	TLR7/8	Nivolumab	PD-1	I/II	Melanoma; merkel cell carcinoma; breast cancer; renal cell carcinoma; colorectal cancer
NCT02880371	ARRY-382	CSF1R	Pembrolizumab	PD-1	II	Advanced solid tumors
NCT03153410	IMC-CS4	CSF1R	Pembrolizumab	PD-1	I	PDAC
NCT02526017	FPA008 (Cabiralizumab)	CSF1R	Nivolumab	PD-1	I	Advanced solid tumors
NCT03708224	Emactuzumab	CSF1R	Atezolizumab	PD-L1	II	Advanced HNSCC
NCT03184870	BMS-813160	CCR2	Nivolumab	PD-1	I/II	PDAC, CRC
NCT03496662					I/II	PDAC
NCT03767582					I/II	Locally advanced PDAC
NCT03447314	GSK1795091	TLR4	Pembrolizumab	PD-1	I	Advanced solid tumors
NCT03445533	IMO-2125	TLR7/8	Ipilimumab	CTLA-4	III	Metastatic melanoma
NCT02644967	IMO-2125	TLR7/8	Pembrolizumab	PD-1	I/II	Metastatic melanoma
NCT02521870	SD101	TLR9	Pembrolizumab	PD-1	Ib/II	Metastatic melanoma, recurrent HNSCC
NCT03007732					I/II	Solid tumors
NCT03618641	CMP-001	TLR9	Nivolumab	PD-1	II	Melanoma
NCT03507699			Ipilimumab	CTLA-4		
NCT02403271	Ibrutinib	BTK	Durvalumab	PD-L1	I/II	Relapsed or refractory solid tumors
NCT02376699	SEA-CD40	CD40	Pembrolizumab	PD-1	I	Solid tumors
NCT01103635	CP-870, 893	CD40	Tremelimumab	CTLA-4	I	Metastatic melanoma
NCT02760797	R07009879 (Selicrelumab)	CD40	Anti-PD-L1	PD-L1	I	Advanced solid tumors
NCT02665416			Bevacizumab/Vanucizumab	VEGF-A	I	Advanced solid tumors
NCT02953782	Hu5F9-G4	CD47	Cetuximab	EGFR	I	Advanced solid malignancies and colorectal carcinoma

## Data Availability

Not applicable.
